# The RELT Family of Proteins: An Increasing Awareness of Their Importance for Cancer, the Immune System, and Development

**DOI:** 10.3390/biomedicines11102695

**Published:** 2023-10-02

**Authors:** John K. Cusick, Jessa Alcaide, Yihui Shi

**Affiliations:** 1 College of Medicine, California Northstate University, Elk Grove, CA 95757, USA; 2 California Pacific Medical Center Research Institute, Sutter Bay Hospitals, San Francisco, CA 94107, USA

**Keywords:** RELT, RELL1, RELL2, TNFRSF, cancer, apoptosis, inflammation, cytokine signaling, interferon, immune cells

## Abstract

This review highlights Receptor Expressed in Lymphoid Tissues (RELT), a Tumor Necrosis Factor Superfamily member, and its two paralogs, RELL1 and RELL2. Collectively, these three proteins are referred to as RELTfms and have gained much interest in recent years due to their association with cancer and other human diseases. A thorough knowledge of their physiological functions, including the ligand for RELT, is lacking, yet emerging evidence implicates RELTfms in a variety of processes including cytokine signaling and pathways that either promote cell death or survival. T cells from mice lacking RELT exhibit increased responses against tumors and increased inflammatory cytokine production, and multiple lines of evidence indicate that RELT may promote an immunosuppressive environment for tumors. The relationship of individual RELTfms in different cancers is not universal however, as evidence indicates that individual RELTfms may be risk factors in certain cancers yet appear to be protective in other cancers. RELTfms are important for a variety of additional processes related to human health including microbial pathogenesis, inflammation, behavior, reproduction, and development. All three proteins have been strongly conserved in all vertebrates, and this review aims to provide a clearer understanding of the current knowledge regarding these interesting proteins.

## 1. Introduction

Members of the Tumor Necrosis Factor Receptor Superfamily (TNFRSF) are intricately associated with governing critical functions including hematopoiesis, activation and migration of leukocytes, inflammation, cell death, and development [1]. Most TNFRSF members function by binding to ligands of the Tumor Necrosis Factor Super Family (TNFSF); there are currently 29 TNFRSF members and 19 TNFSF members identified in humans. Initial studies indicated that TNFRSF members trimerize in response to binding to trimeric TNFSF ligands, yet TNFRSF members are also capable of oligomerizing in the absence of ligand [2,3], and it should be noted that TNFRSF members are capable of binding ligands distinct from TNFSF members [4]. Most TNFRSF members can be classified into receptors that either activate apoptosis through characteristic intracellular “death domains”, activate additional signal transduction pathways through binding TRAFs (Tumor necrosis factor Receptor Associated Factors), or function as decoy receptors that downregulate cell responses to ligands. Modulation of TNFSF–TNFRSF signaling has been approved for the treatment of rheumatoid arthritis, psoriasis, inflammatory bowel disease, cancer, and other diseases [5,6]. This review highlights the TNFRSF member RELT (Receptor Expressed in Lymphoid Tissues), and its two paralogs RELL1 and RELL2 (RELT like 1 and 2, respectively), as there has been a recent increase in the number of reports implicating these three proteins in diverse processes affecting human health including cancer and immune system-related disorders.

## 2. Discovery, Chromosomal Locations, and Single Nucleotide Polymorphisms (SNPs)

RELT was identified [7] by searching the expressed sequence tagged (EST) database for homologies to the Cys-rich extracellular domain (ECD) of the TNFRSF member OX40. The *RELT* gene is encoded on chromosome 11 (11q13.4) and encodes for a Type I transmembrane protein with an ECD that has two Cys regions characteristic of binding TNFSF members, one complete and one incomplete [7]. The ECD is most homologous to OX40, DR3, and the LTβ receptor. RELT is an orphan receptor that did not bind any of the known TNFSF ligands from mice or humans in a previous study [8]. RELL1 and RELL2 were subsequently identified by searching the GeneBank EST database for homologs of RELT. RELL1 and RELL2 are 40% identical at the amino acid level and contain 32% and 27% amino acid identity with RELT, respectively [9]. Homology between the three proteins is strongest within the transmembrane intracellular domains (ICDs). The term “RELT family proteins” (RELTfms) was coined to describe the three proteins due to their homology and their ability to physically interact and co-localize with each other at the plasma membrane [9]. The *RELL1* gene is located on chromosome 4 (4p14) and the *RELL2* gene is located on chromosome 5 (5q31.3). All three genes for RELTfms are strongly conserved in evolution, with each containing orthologs in amphibians, reptiles, birds, and fish.

Interestingly, RELL1 expression is influenced in trans by the single nucleotide polymorphism (SNP) rs7487683, which is predicted to introduce a missense mutation into RAD52 [10]. RAD52 is a protein important for homologous recombination and to resolve double-stranded DNA breaks. RAD52 serves as a potential target for the treatment of BRAC-deficient breast cancer, as RAD52 loss is tolerated much better by healthy human cells than BRAC-deficient cells [11]. Two SNPs (rs3741148 and rs7952686) that influence RELT expression are in close proximity downstream of the RELT gene, in non-coding regions of the adjacent FAM168A transcript [10]. It is likely that these SNPs impact RELT expression by impacting the transcriptional machinery on the neighboring RELT gene, though a direct effect by the FAM168A transcript cannot be excluded.

## 3. Transcription and Transcriptional Regulation

### 3.1. Expression of RELT mRNA

RELT was named for its high levels of mRNA expression in cells and tissues associated with the hematopoietic system [7]. Northern blotting detected a 2.6 kb transcript expressed most prominently in the spleen, lymph node, peripheral blood leukocytes (PBLs), and bone marrow. The transcript was also detected at moderate levels in the fetal liver, colon, testis, and skeletal muscle, yet barely detected in other tissues such as the heart, brain, placenta, lung, small intestine, and kidney. Of the transformed cell lines originally tested, *RELT* mRNA was most abundant in cells of hematopoietic origin, including T cells (MOLT-4), B cells (RAJI), and myeloid cells (HL-60, K-562). RELT was also abundantly transcribed in cervical cancer cells (HeLa) yet was not abundantly expressed in colorectal (SW480), lung (A549), or melanoma (G361) cells. Gene expression data from the ARCHS^4^ platform confirm that the most consistently elevated expression of RELT is in cell lines of lymphocyte origin, yet RELT is also expressed in many other cell lines (Supplementary Figure S1). Data from the Human Protein Atlas (HPA) confirm that *RELT* mRNA is expressed at highest levels in the bone marrow, lymphoid tissues, and PBLs, yet also indicated that *RELT* is transcribed at moderate levels in muscle, neuronal cells, glial cells, trophoblasts, and at lower or barely detectable levels in other tissues such as the lung and gastrointestinal tract. RELT mRNA was similarly detected at high levels in the hematopoietic system of the mouse [12]. RELT expression in the spleen and lymph nodes was not dramatically decreased in mice lacking either FOXN1, RAG2, or NOD [12], suggesting that myeloid cells and non-leukocytes in lymphoid tissue also express RELT. In situ hybridization demonstrated RELT expression in the ameloblasts and odontoblasts of developing mouse teeth [13], consistent with RELT being required for proper enamel formation. Gene expression data from the ARCHS^4^ platform confirms high levels of RELT expression in the hematopoietic system, yet RELT is also expressed in other tissues such as the ovaries, whereas expression of RELT is minimal in the islets of the pancreas (Supplementary Figure S2).

The Alliance of Genome Resources indicates that RELT is detected in developing zebrafish (*Danio rerio*) at the gastrulation stage, approximately 5 h after fertilization. *RELT* mRNA is expressed in Kupffer’s vesicle and axis, indicating that it may be important for establishing the left–right body axis in vertebrate embryos [14]. RELT is expressed in *Xenopus laevis* embryos by approximately 9 h after fertilization, with expression most predominant in adult brain, testis, and muscle. Expression in hematopoietic tissues was not determined in this study, except for the spleen, which exhibited moderate levels of expression [15]. RELT is expressed by 8 days post-conception in developing mice and the highest levels of expression in the adult were in the central nervous system. Interestingly, data indicate that expression of RELT may be more widely distributed in mice than in humans, as in addition to the hematopoietic system, RELT expression was detected in most tissues, including the nervous system and the female reproductive tract [16]. Thus, although RELT is expressed predominantly in hematopoietic tissues, its expression pattern indicates it functions to influence other processes such as development and reproduction, as will be discussed.

### 3.2. Expression of RELL1 mRNA

RELL1 is expressed relatively ubiquitously at the mRNA level, with highest levels detected in the testis, placenta, and in various organs as detected by Northern blotting [9]. The HPA reports the highest *RELL1* transcription is in the bone marrow, placenta, PBLs, and adipose tissue. Gene expression data from the ARCHS^4^ platform confirm relatively ubiquitous expression of RELL1 in cell lines and tissues (Supplemental Figures S3 and S4), though its expression is lower in some cell lines and tissues such as HELAT4 cells and the peripheral nervous system, respectively. The near-ubiquitous expression of RELL1 implies that it likely possesses functions independent of RELT, whose expression is much more tissue restricted. Hepatic *RELL1* mRNA expression exhibits a circadian pattern in mice in a manner likely regulated by miR-122 as livers from mice treated with antisense oligonucleotides targeting miR-122 exhibited elevated levels of mature *RELL1* mRNA. The 3′ untranslated region of *RELL1* mRNA contains a binding site for miR-122, consistent with hepatic RELL1 transcription being controlled by miR-122, an important regulator of liver expression and function. Furthermore, hepatic tissue from mice lacking the transcription factor Rev-erbα (also known as NR1D1) exhibit suppressed levels of *RELL1* mRNA, yet not heterogeneous RNA, suggesting that Rev-erbα influences the circadian rhythm of RELL1 expression by a post-transcriptional mechanism. Further studies are needed to elucidate the significance of circadian RELL1 expression in hepatocytes. Multiple lines of evidence indicate that RELL1 expression is influenced by interferon signaling as will be discussed in relation to regulation of virus infection (Section 10.2). The *RELL1* gene was expressed at the earliest stages of embryonic development in mice (TS1, within 2 days) and was expressed relatively ubiquitously in the adult mouse [16].

### 3.3. Expression of RELL2 mRNA

The expression of *RELL2* mRNA is more tissue restricted than RELL1. Like RELT, *RELL2* mRNA is expressed predominantly in hematopoietic tissues such as the thymus and spleen and is also expressed in immune-privileged sites such as the testes, brain, and placenta [9]. *RELL2* mRNA expression in the HPA was reported to be predominant in various regions of the brain, including the cerebral cortex and medulla, along with high amounts in the parathyroid and pituitary glands, the placenta, testis, hematopoietic tissues, and PBLs. Gene expression data from the ARCHS^4^ platform confirm that RELL2 expression is highest in cell lines of the hematopoietic system whereas very little RELL2 expression is found in certain tissues such as skeletal muscle (Supplemental Figures S5 and S6). Expression of the *RELL2* gene was first detected in mouse embryos at the TS12 stage (E7.5–8.5), and expression in the adult mouse was highest in the nervous system yet was also present in other tissues such as developing enamel [17] and the reproductive and hematopoietic systems [16].

### 3.4. Functional RNA Molecules Expressed from RELTfm Genes

There has been an increasing awareness of the importance of functional RNA molecules in human health and disease, such as circular RNA (circRNA), miRNA, and long noncoding RNA molecules. CircRNA molecules are alternative non-coding, single-stranded, RNA products of a primary transcript that can serve to compete, or “sponge”, for inhibitory miRNA molecules or RNA-binding proteins required for mRNA processing. Competing endogenous RNAs (ceRNAs) transcripts regulate each other at the post-transcriptional level by competing for shared miRNAs. Circ-RELL1 is a circRNA (has_circ_00022194) that consists of exons 5 and 6 from the *RELL1* gene and is upregulated in human umbilical vein endothelial cells (HUVECs) when cells were treated with oxidized LDL [18]. A distinct circRNA RELL1 molecule consisting of exons 4, 5, and 6 was identified that is secreted in exosomes and appears to be protective against gastric cancer [19]. Long noncoding *RELL2* RNA transcripts were identified in extracted intrahepatic cholangiocarcinoma samples [20], yet the significance and physiological function of these RNA molecules is not known.

## 4. Protein Structure, Expression, and Localization

### 4.1. RELT Protein Structure

RELT encodes for a 430 amino acid protein type I transmembrane protein. Initial attempts to express recombinant RELT in HEK-293 (293) cells identified both the expected 46 kDa protein and a smaller protein with an amino terminus corresponding to amino acid position 131 of full-length RELT (beginning with N-terminal sequence GVEV…). These results indicate that a truncated form of RELT is produced by proteolytic cleavage, alternative splicing, or alternative start site selection for transcription or translation [7]. RELT expression at the protein level was most abundant in tissues of the hematopoietic system and [21] a 30 kDa proteolytic fragment was observed in PBLs and other tissues, approximately the same size as the size produced from a predicted cleavage site in the ECD [7]. Interestingly, cleavage of the ECD domain of RELT is necessary for developing ameloblasts [22] as will be discussed. The predicted transmembrane domain (TMD) spans from amino acids 156–191 and the HPA predicts at least two intracellular alpha helices. RELT is predicted to contain a highly disordered structure at its carboxy terminus, indicating the carboxy terminus may fluctuate and adopt different conformations dependent on either post-translational modifications or binding by other proteins [23]. Intracellular sequences of RELT proximal to the membrane are likely important for protein folding and stability, as deletion mutant constructs of RELT lacking amino acids 191–252 were very faintly detected by Western blotting in contrast to other deletion mutants, indicating that RELT constructs lacking amino acids 191–252 were likely degraded in the endoplasmic reticulum due to their inability to fold properly [21]. Importantly, RELT lacks the conserved intracellular “death domain” typical of other apoptosis inducing TNFRSF members, such as Fas and TNFR1.

### 4.2. RELL1 and RELL2 Protein Structure

**Figure 1:**
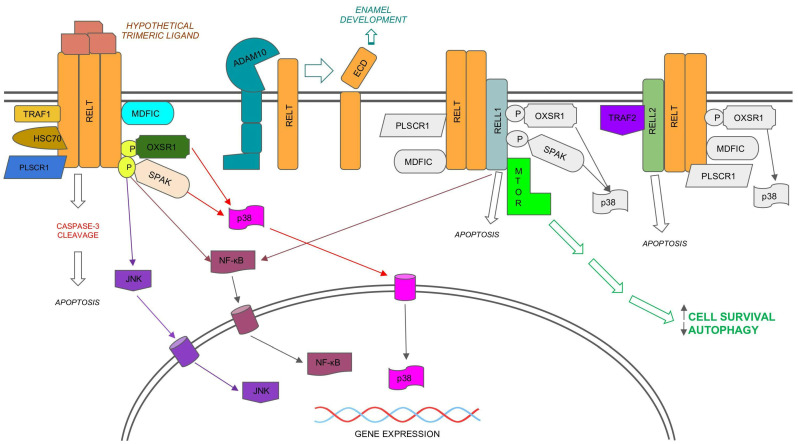
Model for signaling by the RELT family members (RELTfms), RELT, RELL1, and RELL2. Note: RELTfms bind each other, yet it is not yet known if they function as trimers, and it is not known whether RELT binds to an unidentified ligand. The possibility that RELL1 and RELL2 function independently of RELT cannot be excluded. Protein binding partners of RELT that also bind RELL1 or RELL2 (e.g., PLSCR1 [25], MDFIC [23], OXSR1 [9], and SPAK [27,28]) are only shown in color for RELT for simplicity. Unique binding partners to either RELL1 or RELL2 are shown in color. Similarly, the activation of p38 by RELTfms [21,27] is only shown in color for RELT. Activation of NF-κB has been demonstrated for both RELL1 [28] and RELT [7,29]. The activation of the JNK pathway has only been shown for RELT [27]. Not shown are additional localizations reported for RELTfms, including intracellular vesicles [9,25] and the nucleus for RELT, as predicted by the Human Protein Atlas (HPA). The HPA predicts that RELL1 is located at both the plasma membrane and is associated with microtubules, while it predicts that RELL2 localizes to intracellular vesicles. Please note that only known activities of RELTfm proteins are shown. Not shown are activities reported for circular RNA molecules expressed from the *RELL1* gene, including pro-inflammatory functions [18] and the ability to both promote apoptosis and inhibit autophagy [19].

RELL1 and RELL2 are also Type I transmembrane proteins with short sequences in their ECD in comparison to other TNFRSF members, and neither RELL1 nor RELL2 contains extracellular Cys-rich domains used to bind TNFSF ligands (Figure 1). RELL1 encodes a 271 amino acid-long 29.3 kDa protein that appears to be subjected to significant post-translational modifications, as recombinant RELL1 migrates at a position larger than its predicted molecular weight, with multiple bands apparent [9,23,24,25]. RELL2 encodes a 303 amino acid-long 32.4 kDa protein. Both RELL1 and RELL2 are predicted to contain disordered sequences in their carboxy-terminal tails, like RELT, suggesting that the carboxy-termini of RELTfms may adopt multiple conformations depending on either post-translational modifications or the binding of other proteins [23].

### 4.3. Co-Immunoprecipitations of RELTfms and Localization

All RELTfms bind each other as demonstrated by co-IP experiments, and co-expression of recombinant RELTfms resulted in co-localization of these proteins at the plasma membrane of COS-7 cells [9]. Although RELT co-localizes with either RELL1 or RELL2 at the plasma membrane, expression of recombinant RELT by itself resulted in predominant localization within cytosolic compartments in either COS-7 cells [9] and 293 cells [23,25]. Endogenous murine RELT (TNFRSF19L) was observed to be expressed at the plasma membrane in the human lung tumor cell line H226 [26]. Interestingly, the HPA predicts that RELT localizes to the nucleoplasm, based on information using an antibody raised against intracellular residues 204–275 as an epitope, suggesting that the ICD of RELT may be cleaved, resulting in the truncated carboxy terminus migrating to the nucleus. Expression of recombinant RELL1 and RELL2 individually was observed to localize at the plasma membrane in COS-7 [9] and 293 cells [23]. The HPA confirms that RELL1 is expressed at the plasma membrane, yet also indicates that it is associated with microtubules, whereas this database predicts that RELL2 localizes to intracellular vesicles. Interestingly, one report suggests murine RELL2 (ependolin) is secreted and interacts with the extracellular matrix [17].

## 5. Phenotypes of Mice Lacking RELTfms

### 5.1. Phenotype of RELT Knockout Mouse

Mice lacking RELT exhibit enhanced populations of CD4^+^ T cells, and enhanced CD8^+^ T cell responses to tumor cells, indicating that RELT likely functions in part by suppressing T cell responses [12]. RELT^−/−^ mice are viable and fertile, and contain normal populations of different leukocytes, indicating RELT is not essential for hematopoiesis. *RELT* mRNA expression in CD4^+^ and CD8^+^ T cells was dramatically reduced upon stimulation of T cells with CD3 antibody, and a smaller but significant decrease in *RELT* mRNA was also observed when dendritic cells were stimulated with LPS. Stimulated T cells in RELT^−/−^ mice were characterized by elevated IFN-γ secretion for both CD4^+^ and CD8^+^ T cells and elevated levels of IL-5 and TNF-α secretion from stimulated CD4^+^ cells and CD8^+^ T cells respectively. RELT^−/−^ mice had significantly elevated levels of CD4^+^ T cells in lymphoid organs, a phenotype that was also observed after the transfer of RAG2^−/−^ mice with RELT^+/+^ CD4^+^ lymphocytes, indicating that RELT likely suppresses the homeostatic proliferation of CD4^+^ T cells. A reduced tumor growth rate and an increased number of infiltrating CD8^+^ lymphocytes were observed when MC38 colon adenocarcinoma cells were injected into RELT^−/−^ mice in comparison to control mice, indicating that RELT counters the tumor-killing ability of CD8^+^ cells. Mice harboring B16–10 melanoma cells exhibited a greater number of infiltrating tumor-specific CD8^+^ T cells and a reduced tumor growth rate after being infused with naïve RELT^−/−^ CD8^+^ T cells in comparison to naïve WT CD8^+^ T cells. In contrast, no difference in tumor growth was observed after the adoptive transfer of previously activated CD8^+^ lymphocytes from either a RELT^−/−^ or WT background, supportive of a model in which RELT inhibits the activation of CD8^+^ T cells. Similarly, RELT^+/+^ CD8^+^ T cells exhibited increased tumor antigen-specific activation and tumor growth suppression when transferred into RELT^−/−^ mice versus RELT^+/+^ mice, suggesting that expression of RELT in additional cells besides CD8^+^ T cells also function to inhibit CD8^+^ T cell activation. An increased number of RELT^−/−^ CD8^+^ T cells persisted after antigen stimulation, and no differences in proliferation were observed between CD8^+^ lymphocytes containing or lacking RELT, suggesting that RELT may function in part to promote activation-induced death in T cells. Collectively, these studies indicate that RELT inhibits both CD8^+^ responses against tumors and CD4+ T cell homeostatic proliferation, in addition to suppressing inflammatory cytokine secretion by T lymphocytes. The activation of myeloid-derived suppressor cells (MDSCs) in response to tumor growth was also reduced in mice lacking RELT [12], supportive of a model in which RELT suppresses immune responses against tumors. Therefore, inhibiting RELT expression in T cells is an avenue worth exploring to potentially increase the effectiveness of adoptive cell therapy for tumor patients [12].

### 5.2. Phenotype of RELL1 Knockout Mouse

RELL1 is an important gene for the proper functioning of the nervous system based on phenotypes of mice lacking RELL1. A deletion of exon 4 and the flanking intronic sequence was made in C57BL/6NJ mice to produce a truncated protein [30]. Data available on the International Mouse Phenotyping Consortium www.mousephenotype.org (accessed on 24 September 2023) indicate the most prominent phenotypes of these mice thus tested were in behavior and auditory processing. Both sexes exhibited decreased prepulse inhibition and increased startle reflex behavior, though the increased startle reflex behavior was much more pronounced in males. Mice of both sexes exhibited abnormal auditory brainstem response, yet only female mice exhibited hyperactivity as determined by the open field-center start phenotypic assay. Based on this mouse phenotypic data, RELL1 may be important for several neurological disorders, including attention deficit-hyperactivity disorder, intellectual development disorder, schizophrenia, and deafness. In contrast, no detectable effects of the null RELL1 mutation on mortality, growth, the skeleton, pigmentation, vision, reproduction, metabolism, or the cardiovascular and immune systems were detected. It should be noted that these studies were conducted with a limited number of sterile mice, and that further studies examining the response of knockout mice to infectious pathogens are needed to better understand the importance of RELL1 to the immune system.

## 6. Activation of Signaling Pathways by RELTfms

The understanding of pathways activated by RELTfms remains sparse compared to other TNFRSF members, yet an improved appreciation for the physiological relevance of these genes is beginning to emerge. Two separate groups confirmed that RELT does not bind TRAFS 2–6, though there have been conflicting accounts as to whether RELT binds TRAF1 [7,27]. RELL2, yet not RELT or RELL1, physically interacts with TRAF2 [24], and it remains to be determined if other TRAFs bind either RELL1 or RELL2.

### 6.1. Activation of NF-κB by RELTfms

RELT was originally identified as a protein that upregulates NF-κB in 293 cells in a manner that was independent of TRAF1 [7], yet a separate study using 293 cells did not find RELT overexpression increased the activation of NFκB [27]. RELT expression was correlated with increased cell proliferation and nuclear localization of NF-κB in esophageal cancer cell lines [29]. Inhibitors of NF-κB negated the pro-survival effects of RELT expression in esophageal cancer cells, and knockdown of RELT expression in mice decreased the nuclear localization of NF-κB in vivo. The ability of RELT to activate NF-κB in esophageal cells is consistent with the finding that RELT is associated with NF-κB based on gene enrichment analysis of the TCGA database [29]. Multiple lines of evidence indicate that both *RELL1* RNA and RELL1 protein are pro-inflammatory and activate NF-κB. A circular RELL1 RNA (circRELL1) composed of exons 5 and 6 (has_circ_0002194) was the circular RNA most upregulated by exposure of HUVEC cells to oxidized LDL [18]. CircRELL1 promotes inflammation by relieving the inhibition (i.e., sponging) of the anti-inflammatory microRNA miR-6873-3p. Knockdown of circRELL1 was associated with reduced p65 nuclear localization and reduced expression of MyD88, ICAM, and VCAM. MyD88 is a powerful activator of NF-κB, while ICAM and VCAM are adhesion molecules that promote the extravasation of neutrophils into inflamed tissues. These results are supportive of a model in which the pro-inflammatory activity of circRELL1 contributes to atherosclerosis and heart disease. RELL1 protein expression is pro-inflammatory and increases the activation of NF-κB and AP-1 in murine macrophages in response to *M. tuberculosis* infection [28]. Although the authors of this study were focused on the RELL1 protein, it is not clear if circRELL1 also contributed to the reported observations.

NF-κB is a general term used to describe a family of dimeric transcription factors and the importance of NF-κB for both inflammation and survival of cancer cells is well documented [31,32]. Proteins utilized to make active NF-κB transcription factors include p100, p105, RelA, RelB, and RelC, and the Rel homology domain of NF-κB proteins enables dimerization, nuclear localization, and DNA binding. NF-κB dimers are sequestered in the cytosol by inhibitor of κB proteins (IκB). NF-κB activation requires upstream signals to activate IκB kinases (IKKs). Phosphorylation of IκB proteins by IKKs result in the ubiquitination and degradation of IκB proteins, thus liberating NF-κB dimers and permitting their entry into the nucleus. The canonical pathway involves a dimer of p50 and RelA (p65) being liberated by IκB degradation, while activation of alternative pathways requires proteolytic cleavage of p105 and p100 to produce fragments, p50 and p52, respectively, that form active dimers of p50/p50 or p52/RelB. Nuclear NF-κB induces the expression of many inhibitors of apoptosis, including proteins that inhibit the activation of caspases (FLIP) and proteins that inhibit caspases directly (c-IAPs) [33]. Activities of multiple TNFRSF members are dependent on the activation of NF-κB, including the stimulus of bone metabolism by RANK when bound to RANKL [34] and the pro-inflammatory effects of TNFR1 when bound by TNFα [35]. Identifying inhibitors of NF-κB is of great interest for the treatment of cancer and inflammatory diseases, due to the ability of NF-κB to promote the survival of cancer cells in response to chemotherapeutic agents and to the pro-inflammatory effects of NF-κB signaling [32,36].

### 6.2. Association of RELTfms with Interferon Signaling

Multiple lines of evidence strongly implicate both RELT and RELL1 with interferon (IFN) signaling. A genome-wide siRNA microarray screen of genes that influenced the survival of *M. tuberculosis* in infected monocyte Thp1 cells identified RELL1 as a protein that is downregulated by IFN-γ [37]. Subsequent studies confirmed RELL1 expression is significantly downregulated when a bone-marrow-derived murine macrophage cell line (RAW264.7) was either treated with IFN-γ or infected with *M. tuberculosis*, as determined by both RT-PCR and Western blotting [28]. A separate study used both microarray and qRT-PCR from mouse lymphocytes to demonstrate that *RELL1* mRNA expression is reduced in Treg cells lacking the IFN-α/β receptor [38], indicating RELL1 expression is activated by Type I IFN signaling in Tregs. Although the effects on distinct cell lines may differ, RELL1 is suppressed by IFN-γ signaling in two separate macrophage cell lines and is suppressed by the lack of Type I IFN signaling, indicating that Type I and Type II interferons have opposite effects on RELL1 expression. As mentioned previously, CD4^+^ and CD8^+^ T cells from mice lacking RELT exhibit elevated levels of IFN-γ secretion, implicating RELT as a protein that suppresses IFN-γ secretion from T-cells. However, it is worth noting that RELT and the IFN gamma receptor were both significantly upregulated in gastric cancer patients versus control subjects [39], so RELT may not act antagonistically towards IFN-γ signaling in all settings.

## 7. Activation of p38 and JNK MAPK Pathways by OXSR1 and SPAK Kinases

Phosphorylation of RELT by either of the closely related kinases OXSR1 (Oxidative Stress Response 1, also known as OSR1) and SPAK (STE20/SPS1-related, proline alanine-rich kinase, also known as STK39) kinases results in activation of the p38 pathway (Figure 1) in 293 s [21,27]. Phosphorylation of RELT by SPAK results in the activation of the JNK pathway in 293 s. A kinase dead mutant of SPAK disrupts the ability of RELT to activate p38 and JNK pathways [27], and a kinase dead mutant of OXSR1 disrupts the ability of RELL1 or RELL2 to activate p38 [21]. Furthermore, the ability of RELTfms to activate the transcription factor AP-1, a downstream target of both JNK and p38, has been shown for RELT in 293 s [27] and RELL1 in activated macrophages [28]. Knockdown of RELL1 expression reduced activation of p38, JNK, NF-κB, and ERK in a mouse macrophage cell line (RAW264.7) infected with *M. tuberculosis* [28]. Although physical interaction between RELL1 and SPAK was confirmed in the extracts of these activated macrophages, it was not demonstrated conclusively that interaction with SPAK was required for RELL1 activation of these MAPK pathways. Collectively, these results establish that RELTfm activation of p38 is dependent on phosphorylation by the OXSR1/SPAK kinases, and that RELT also activates the JNK pathway in a manner that is dependent on SPAK.

SPAK and OXSR1 recognize their targets through the binding motif [S/G/V]RFx[V/I/]xx[V/I/T/S]xx, and RELTfms are the only TNFRSFs to contain this motif [40], present as GRFRVxxI/V in RELTfms. SPAK does not bind or phosphorylate a RELT mutant with a F350A mutation disrupting the GRFRVxx motif, and this mutant form of RELT is unable to activate p38 and JNK pathways in 293 cells [27]. Consistent with these results, deletions from the intracellular domain (ICD) of RELT that lack the GRFRV motif have less activation of p38 than full-length RELT [21]. OXSR1 and SPAK are both Serine/Threonine kinases strongly conserved in evolution and exert a profound influence over ion transport, cell volume regulation, cancer, and migration [41]. The amino acid sequences of these two paralogs are 67% identical overall, with 89% identity in the catalytic region [41]. Both kinases autophosphorylate themselves and are expressed nearly ubiquitously, with particularly strong levels of SPAK expression in the brain. Both kinases contain a caspase cleavage site and SPAK contains a nuclear localization sequence. Although both kinases are largely expressed in the cytosol, SPAK lacking the caspase cleavage site at the carboxy terminus translocates to the nucleus [42].

The p38 and JNK mitogen-activated protein kinases (MAPKs) are activated in response to extracellular or intracellular stimuli and exert a profound influence on cell growth, migration, division, differentiation, and death through the phosphorylation of downstream target [43]. While the ERK kinases are predominantly associated with pro-tumorigenic properties such as cell proliferation and survival, both JNK and p38 possess both pro- and anti-tumorigenic properties, that are likely to depend on the type of cell, duration, and magnitude of signaling, in addition to input from other signal transduction pathways.

The p38 family of kinases is composed of four closely related homologs, α, β, γ, δ, although the α isoform has been most extensively studied, is most ubiquitously expressed, and will be described here unless otherwise noted. p38 is a MAPK that is activated in response to multiple cellular stresses and, when active, can phosphorylate targets leading to alterations in cell migration, division, migration, death, and inflammation [44]. Mice lacking the upstream kinases MKK3 and MKK6 show a higher tendency to develop tumors, which is consistent with the ability of p38 to inhibit the development of tumors. However, once tumors are established, p38 may in fact assist the progression of advanced cancers [45], as p38 inhibition prevents metalloproteinase MMP-9 expression. p38 also promotes DNA repair, and p38 inhibitors are potential treatments for cancer patients receiving chemotherapy, although the pleiotropic effects of p38 have posed a challenge for p38 inhibitors in clinical trials for cancer treatment thus far. The pleiotropic effects of p38 might also explain the rare incidence of p38 mutations in cancer, despite its established ability to assist the progression of advanced tumors. p38 can also induce pro-inflammatory cytokine secretion, and considering the established correlation between inflammation and cancer, it is not surprising that studies have implicated the inflammation induced by differing isoforms of p38 as being pro-tumorigenic [46,47].

The JNK family of kinases are ubiquitously expressed and consist of three primary isoforms (JNK1, JNK2, and JNK3) that are activated by the upstream kinases MKK4 and MKK7 [48]. JNK activates a group of transcription factors known as AP-1 (activator protein 1), which exist as homodimers or heterodimers of the Jun, Fos, ATF, or MAF protein families. It is somewhat contradictory whether JNK kinases are pro- or anti-apoptotic. JNK can promote apoptosis by phosphorylating and stabilizing p53 in response to cell stress [49]. Similarly, JNK can inactivate the anti-apoptotic protein Bcl-2 through phosphorylation. Conversely, JNK possesses anti-apoptotic activities, for example by blocking Caspase-9 activation [50], or by liberating the anti-apoptotic protein Bcl-XL from Bad [51]. There is extensive crosstalk between parallel MAPK pathways, including p38 and JNK. For example, p38 can activate the phosphatase that inhibits JNK in response to UV light, protecting mouse fibroblasts from JNK-mediated apoptosis [52], yet higher levels of JNK activation can overcome this inhibition and lead to cell death [53].

## 8. Activation of Cell Death Pathways

### 8.1. Activation of Apoptosis by RELTfms

Apoptosis is an active, energy-requiring process that results in cell death without the release of inflammatory cellular compartments into the environment. TNFRSF members can induce apoptosis in response to external ligands by the extrinsic pathway of apoptosis as has been reviewed elsewhere [54,55]. RELTfms are unusual in that they can induce cell death despite not containing the characteristic death domain found in other death-promoting TNFRSF members. Overexpression of RELTfms in 293 cells results in apoptotic cell rounding, DNA fragmentation [24], and Caspase-3 cleavage [25]. Presumably, overexpression of RELTfms induces clustering of the receptor to form trimers in the absence of ligand, as demonstrated for TNFR1 previously [56], yet the dependence on oligomerization of RELTfms for inducing death has not been proven. The magnitude of RELT-induced death in 293 cells versus empty vector control was enhanced in media containing 2% FBS versus 10% FBS and the time course of death induced by RELT overexpression as measured by both cell rounding and DNA fragmentation was delayed by approximately a day in comparison to TNFR1 [24]. Deletion mutant analysis of RELT indicated that the carboxy-terminus of RELT is required for death [21], though it is not clear if a novel death domain exists at the carboxy-terminus, or whether multiple domains in the ICD are required for cell death. Unlike TNFR1, the ability of RELT to induce cell rounding in 293 cells was not inhibited by CrmA, or by dominant negative mutants of either FADD or Caspase-8 [21]. CrmA is a viral inhibitor of caspases that strongly blocks activation of apoptosis by additional TNFRSF members such as Fas [57] by inhibiting the proteolytic activity of Caspases 1, 6, and 8. In contrast, CrmA exhibits minimal inhibition of Caspase-3 in vitro [58]. Collectively, these data suggest RELT activates apoptosis by a mechanism that is independent of Caspase-8 or Fadd in 293s.

The ability of RELTfms to induce death in different cell types is not universal, and both the mechanism of RELTfm-induced death, and the physiological implications of RELTfms-induced death on human health and disease need to be explored further. Data utilizing the cell line AR42J indicate RELT promotes apoptosis after stimulation of pancreatic acinar cells with caerulein, a peptide utilized to induce pancreatitis in mouse models [59]. The cells were sorted into cells undergoing apoptosis versus oncosis, and RELT protein was exclusively associated with the apoptotic population, suggesting that RELT may participate in the induction of apoptosis of acinar cells in pancreatitis. RELT has also been indirectly implicated in the apoptosis of trophoblast cells, as deletion of the transcription factor ZBED1 resulted in both upregulation of RELT mRNA expression and apoptosis of trophoblasts [60]. RELT mRNA expression was downregulated in chicken splenic lymphocytes treated with the protective saponin Rg1 prior to treatment with hydrogen peroxide versus hydrogen peroxide alone, as determined by both transcriptome analysis and qRT-PCR [61]. Similarly, Jun and Fos expression was also downregulated by the protective Rg1 in this study, suggesting that Rg1 may protect chicken splenic lymphocytes from oxidative stress-induced apoptosis in part by downregulating RELT and JNK, which is of interest considering that RELT is phosphorylated by kinases that are responsive to oxidative stress [9,27]. Conversely, RELT is implicated as an oncogene that inhibited apoptosis in esophageal squamous cell carcinoma (ESCC), as determined by AV/PI staining, and Western blotting of Survivin and cleaved Caspase-3 [29]. The functions of RELT may be cell- and tissue-dependent, as differential post-translational modifications of RELT and integration with tissue-specific proteins may influence the physiological function of RELT in a tissue-dependent manner.

RELL1 and RELL2 also induced death when overexpressed in 293 cells, yet curiously, co-expression of RELL2 with either RELL1 or RELT decreased the amount of DNA fragmentation in comparison to overexpression of individual RELTfms [24]. This interesting result may indicate that heterotrimeric RELT family member signal transduction complexes may result in different signaling outcomes in comparison to homotrimeric signaling complexes. It should be emphasized that it has not been proven that RELT functions as a trimer when initiating signaling pathways, yet it is intriguing that RELL2, but not RELT or RELL1, binds to TRAF2, demonstrating the possibility that heterotrimeric complexes of RELTfms may have distinct signaling outcomes in comparison to homotrimeric RELT complexes. RELL2 has been strongly implicated in promoting apoptosis in the triple-negative breast cancer cell line MDA-MB-231 (231). The saponin Polyphyllin VI, derived from the medicinal plant *Paris polyphylla*, upregulates the expression of RELL2 in 231 cells by relieving the inhibitory effects of miR-18a on RELL2 expression [62]. Polyphyllin VI treatment induced apoptosis in 231 cells, implicating that upregulation of RELL2 in 231 cells likely causes apoptosis, though this saponin potentially exerts effects on additional signaling pathways besides RELL2 and the ability of RELL2 to induce apoptosis in 231 cells has not been clearly established. Conversely, RELL1 expression in activated macrophages was associated with inflammation, and a correlation between RELL1 and apoptosis was not observed when macrophages were stimulated by either *M. tuberculosis* infection or with TLR2 agonists [28]. However, expression of an untranslated circRELL1 increased apoptosis in gastric cancer cells in both organoids and in mouse xenografts as measured by TUNEL staining [19]. It therefore appears that the effects of the *RELL1* gene on apoptosis may differ depending on the cell or tissue type, and we cannot discount potentially differing effects of RELL1 protein versus circRELL1 on cell viability.

### 8.2. Autophagy Inhibition by RELL1

Multiple reports indicate both RELL1 protein and circRELL1 RNA inhibit autophagy. Autophagy is the process by which cells can engorge organelles and damaged proteins through the formation of a catabolic phagosome [63]. Autophagy can result in the death of pro-tumorigenic cells, yet conversely can provide nutrients for anoxic tumor cells to assist their survival [64]. RELL1 protein expression in a mouse macrophage cell line was associated with the inhibition of autophagy and increased survival of *M. tuberculosis* [28]. RELL1 expression inhibited autophagic flux, as determined by conversion of LC3I to LC3II in both macrophage-infected macrophages, as well as HEK-293T cells. RELL1 binds mTOR in 293 cell extracts and RELL1 expression results in increased phosphorylation of the mTOR substrate p70S6K, indicating that RELL1 likely inhibits autophagic flux by binding and activating mTOR (Figure 1). Untranslated circRELL1 also possesses anti-autophagic function in gastric cancer cells. Expression of circRELL1 acts as a sponge for miR-637 [19], which likely relieves repression of anti-autophagic proteins such as EPHB3. Expression of circRELL1 and miR-637 appear to counteract each other with regards to EPHB3 expression and autophagy, as measured by LC3 cleavage and autophagic flux.

## 9. Association of RELTfms with Cancers

Multiple lines of evidence strongly implicate RELTfms as being important for multiple types of cancer, yet data supporting a mechanism for how individual RELTfms function in either a pro- or anti-tumorigenic manner in most cancers are lacking. Both experimental evidence and bioinformatic analysis indicate that correlations of individual RELTfms vary significantly for different types of cancer (Table 1).

### 9.1. Breast Cancer

Current evidence indicates that RELT is a tumor-associated antigen upregulated in breast cancer, although it is unclear whether there is a causal association between RELT and breast cancer. RELT autoantibodies (autoAbs) were identified to be a potential prognostic indicator for the early detection of breast cancer in a study comparing the serum of 87 healthy women and 87 women with varying stages of breast cancer [65]. AutoAbs against RELT represented the most prominent prognostic indicator for breast cancer in this study, as autoantibodies against RELT alone could diagnose breast cancer with a diagnostic accuracy of 71%, though it should be emphasized that separate studies identified autoAbs against other proteins as more predictive of breast cancer [79,80]. Nevertheless, the identification of RELT autoAbs as a potential biomarker for breast cancer in one study is consistent with RELT being either mutated or expressed at higher levels in breast cancer to invoke an immune response. RELT is a member of a 123-gene cluster upregulated when a murine mammary cell line was induced with TGF-β to undergo the epithelial-to-mesenchymal transition (EMT) [66]. Additionally, bioinformatic analysis indicates this gene cluster is also highly expressed in monocyte-derived immune cells, with RELT specifically upregulated 2.2-fold in granulocytes, mast cells, and peripheral macrophages. Bioinformatic analysis revealed the gene cluster was enriched in breast cancer cell lines, with the cluster most significantly linked with cell lines representing basal B, or triple-negative, breast cancer. Lastly, analysis of a microarray data set representing 107 human breast tumors indicated that the gene cluster was most upregulated in biopsies representing triple-negative breast cancer [66].

RELL2 possesses anti-tumorigenic activities against the breast cancer cell lines 4T1 and 231; treatment of these cell lines with the saponin PPVI upregulates RELL2 expression by relieving the inhibition of mir-18a on RELL2 expression [62]. Either expression of recombinant RELL2 or treatment with PPVI suppressed the in vitro migration and invasion of both 4T1 and 231 cells, whereas siRNA directed against RELL2 had the opposite effect. Additionally, administration of PPVI inhibited the growth of 4T1 cells in mice and significantly reduced the number of metastatic lesions in lungs. PPVI also induced both S-phase cell cycle arrest and apoptosis in 231 cells as measured by flow cytometry [62]. These results demonstrate that RELL2 inhibits the migratory and invasive capabilities of breast cancer cell lines ex vivo and suggest that RELL2 prevents tumor growth and metastasis of 4T1 cells in vivo. The ability of PPVI to induce the death of 231 cells provides indirect evidence that RELL2 induces the death of 231 cells, yet both PPVI and miR-18a likely possess additional targets, so these results must be interpreted with caution.

### 9.2. Esophageal Squamous Cell Carcinoma (ESCC)

RELT exhibits pro-tumorigenic activity in ESCC, both ex vivo and in vivo, by a pathway that requires NF-κB [29]. RELT expression increased cell viability and decreased apoptosis in ESCC cells. Treatment of ESCC cells with the NF-κB inhibitor PDTC blocked the ability of RELT to protect ESCC cells from death, implying that RELT serves as an oncogene in ESCC cells by upregulating NF-κB. Importantly, knockdown of RELT expression in ESCC cells prior to xenografting the cancerous cells in nude mice suppressed tumorigenic capabilities of the ESCC cells in vivo. Analysis of xenografted tumors confirmed that RELT expression upregulated NF-κB and inhibited apoptosis in this in vivo model. Consistent with these findings, mining of the TCGA database indicates that RELT is expressed at higher levels in ESCC in comparison to non-malignant tissue, and higher expression of RELT was associated with a worse prognosis as determined by Kaplan–Meier survival analysis. Finally, RELT analysis of paired cancerous and non-cancerous tissues from ESCC patients revealed higher levels of RELT expression in ESCC as measured by qRT-PCR, Western blotting, and IHC [29].

In contrast to RELT, RELL2 appears to play a protective role against ESCC. RELL2 nascent RNA is subjected to editing by the adenosine deaminase enzyme ADAR2, and this editing influences the ability of RELL2 to exert an anti-tumorigenic effect in ESCC cell lines [67]. ADAR2 binds a dsRNA region of *RELL2* nascent transcript between exon 3 and a polypyrimidine tract, and this binding by ADAR2 results in exon 3 skipping. The skipping of exon 3 prevents the synthesis of a functional RELL2 protein in both 293 cells and the ESCC cell line EC109, as a stop codon is generated in exon 6, and the *RELL2* transcript lacking exon 3 is degraded by nonsense-mediated decay. Overexpression of RELL2 containing exon 3 in EC109 cells resulted in a less tumorigenic phenotype, as measured in vitro by foci formation and soft agar colony assay-forming assay. Additionally, expressing full-length RELL2 with exon 3 reduced the tumorigenic potential of EC109 cells in vivo as determined by xenograft tumor formation in mice.

### 9.3. Prostate Cancer

RELT was one of five signature genes that predicted poor prognosis for prostate cancer patients [68]. Patients in the cohort with higher expression of RELT were more likely to have cancers with an immunosuppressive environment, in addition to a higher mutation frequency, higher incidence of metastasis to lymph nodes, and worse prognosis. An additional bioinformatic study demonstrated a correlation of RELT expression with IQGAP3, a protein that influences cytoskeleton dynamics and poor prognosis for prostate cancer patients [69]. Furthermore, this study identified RELT as a member of a 13-gene panel, SigIQGAP3NW, that predicts poor outcomes for prostate cancer. This gene panel was most strongly associated with genes associated with mitosis and chromosome segregation, in particular the genes for Polo-like Kinase 1 (PLK1) and TOP2A. PLK1 and TOP2A are both implicated in cancer and serve as targets for chemotherapy. PLK1 localizes to centrosomes and serves as an early trigger for the G2/M transition [81] while TOP2A is a DNA topoisomerase [82]. SigIQGAP3NW was associated with higher numbers of Tregs, Th2 lymphocytes, and mesenchymal stem cells, all of which promote resistance of tumors to immune surveillance. RELT expression in prostate cancer correlated significantly with Tregs, dendritic cells, myeloid-derived suppressor cells, and dendritic cells, indicating that RELT may promote an immunosuppressive environment. SigIQGAP3NW expression also serves as an accurate predictor of poor responses to chemotherapy, and given the strong association of this gene signature with the chemotherapy targets PLK1 and TOP1, targeting PLK1 and TOP1 may be a strategy to treat cancer patients expressing high levels of the signature SigIQGAP3NW, including high levels of IQGAP3 and RELT.

### 9.4. Glioma and Glioblastoma

RELL1 is upregulated in gliomas and is a poor prognostic indicator for survival, as bioinformatic analysis revealed that RELL1 is expressed more prominently in metastatic brain tumors that have a poor prognosis [70]. RELL1 expression was correlated with an immunosuppressive environment and inversely correlated with NK cell activity in both the CGGA and TCGA databases. A separate study identified an intracellular N255D mutation of RELL1 exclusive to glioblastomas yet not normal astrocytes [71]. Interestingly, N255 is flanked by several Thr residues (221, 253, and 261) and Ser residues (209, 212, 237, 244, 247, and 249) in the immediate vicinity of the binding site for the Ser/Thr kinases and paralogs OXSR1 and SPAK (226–233). The RELL1 protein is likely phosphorylated multiple times by OXSR1/SPAK or other kinases, as Western blots have consistently shown a banding appearance for RELL1 [9,23,24,25]. It is interesting to speculate whether a negative charge introduced by the phosphorylation of RELL1 results in a similar conformation as produced by the N255D mutation, resulting in a pro-tumorigenic form of the protein. In contrast to the upregulation of RELL1 in glioblastoma, bioinformatic analysis indicates that RELL2 is expressed at lower levels in glioblastoma multiforme (GBM) and low-grade gliomas (LGG) versus normal non-malignant tissues [83].

### 9.5. Gastric Cancer

The *RELL1* gene is also transcribed to produce a circRNA that is secreted in exosomes and functions to inhibit the progression of gastric cancer [19]. Analysis of bioinformatic databases indicates that lowered circRELL1 expression is associated with poor prognosis in gastric cancer. Co-culture of cells secreting exosomal circRELL1 inhibited the proliferation of both cancerous and non-cancerous gastric epithelial cell lines. Furthermore, gastric cancer cell lines expressing circRELL1 exhibited reduced tumorigenic capacity when injected into nude mice. Conversely, inhibition of circRELL1 expression resulted in increased viability and growth of gastric cancer cells ex vivo and in vivo. circRELL1 binds and sponges the pro-tumorigenic miR637 and relieves the inhibitory effect of miR637 on other proteins such as EPHB3. Expression of circRELL1 and EPHB3 are associated with decreased autophagy in gastric cancer cells, and since circRELL1 is secreted in exosomes, it has the capacity to inhibit tumorigenic potential in neighboring cells. In contrast to circRELL1, mutations in the RELL1 protein may be pro-tumorigenic for gastric cancer, as the N255D mutation described previously for glioblastoma was also expressed in at least 30% of gastrointestinal tract cancer patients [71].

RELT may serve as a potential biomarker for gastric cancer, as RELT in serum is upregulated approximately 2.7-fold in patients with gastric cancer as measured by both a cytokine antibody array as well as by ELISA [39]. It is not clear whether the upregulation of RELT is associated with tumorigenesis of gastric cancer, or whether it is a byproduct of gastric cancer.

### 9.6. Pancreatic Cancer

RELL2 expression results in increased death and sensitivity of pancreatic ductal adenocarcinoma (PDAC) cell lines to the nucleoside analog gemcitabine [72]. The splicing factor DHX38 promotes RELL2 protein expression by promoting the splicing of an intron (intron 4) that would otherwise promote nonsense mediated decay of the *RELL2* transcript if retained. Bioinformatic analysis indicates RELL2 expression is a good prognostic indicator for PDAC. Bioinformatic analysis by a separate group confirmed that higher RELL2 expression was correlated with increased survival and better patient outcomes for pancreatic adenocarcinoma (PAAD) [83]. It is important to note that two separate groups report that post-transcriptional modifications of *RELL2* nascent RNA modulate the anti-tumorigenic functions of RELL2 in pancreatic [72] and ESCC [67]. It is interesting to speculate whether therapeutics that promote RELL2 transcript stability and translation may help with the treatment of cancers.

### 9.7. Lung Cancer

*RELT* mRNA was found to be upregulated in the lung tumors versus non-malignant tissue of four patients [73]; most (16 out of 19) patients in this study had non-small cell lung cancer (NSCLC). *RELT* mRNA was downregulated 1.9-fold in response to treatment with the anticancer agent romidepsin in at least one patient with lung cancer in this study [73]. Microarray analysis demonstrated that RELT expression is upregulated in a non-small cell lung cancer cell line (A549) treated with the glucocorticoid dexamethasone [84]. RELT was identified to be the receptor for the specific delivery of a peptide-conjugated nanoparticle delivery system of drugs to lung cancer tissue in a mouse model [26]. RELT was upregulated two-fold in mouse lung tumors induced by a K-ras G12D mutation compared to non-malignant tissue [26]. Conversely, analysis of both genomic profiles and RNA from 137 patients indicates that RELT expression is decreased in small-cell lung cancer (SCLC). Furthermore, RELT expression was predicted to be one of the most protective genes from this study [74]. Finally, mutations in the RELL1 protein may be pro-tumorigenic for lung cancer, as the N255D mutation described previously for glioblastoma was also expressed in at least 30% of lung cancer patients [71].

### 9.8. Renal Cancer

RELT expression is most strongly correlated with poor prognosis in renal cancer, with a P score of 1.1 × 10^−10^ https://www.proteinatlas.org/ (accessed on 9 September 2023). RELT expression has a positive correlation with Tregs (R = 0.27) and an inverse correlation with resting mast cells (R = 0.29) in clear cell renal cell carcinoma (ccRCC) [75], suggesting that the negative outcome of RELT expression on RCC patient survival may be in part due to the promotion of a tumor suppressive environment. RELT was one of eight genes that served as an accurate predictor of poor RCC patient survival. This study reported a very strong correlation of RELT expression in RCC with KCNN4, a potassium channel whose expression is upregulated in activated T cells [85]. RELT is a member of a 13-gene panel, SigIQGAP3NW, that predicts poor outcomes for papillary renal cell carcinoma (PRCC) with high confidence [69]. RELT expression correlates strongly with many immune checkpoint blockade (ICBs) targets such as CTLA-4, TGF-β, PD-L2, in many cancers such as renal cell carcinoma [75] and prostate cancer [69], as mentioned previously.

### 9.9. Head and Neck Squamous Cell Carcinoma

RELT expression is suppressed by miR-424-5p in head and neck squamous cell carcinoma (HNSC) patients, and interestingly, RELT expression was also suppressed by a variety of chemotherapeutic agents in HNSC patients [76]. Bioinformatic analysis revealed that RELT expression was positively correlated with several markers associated with immunosuppression, including PD-1, CTLA-4, and PD-L1. Additionally, RELT appeared to be downregulated in naïve B cells and Tregs and upregulated in neutrophils. RELT expression was negatively correlated with overall survival of HNSCC patients.

### 9.10. Cancers of Hematopoietic Origin

RELT was upregulated in the lymph nodes of patients with B cell lymphoma versus normal lymph nodes [23]. RELT was expressed in the malignant infiltrating B cells, as RELT expression co-localized with CD20. The HPA confirms higher levels of RELT expression in diffuse cancers of the immune system, such as B-cell lymphoma, leukemias, and myeloma. This report also characterized the staining of RELT in normal lymph nodes, demonstrating strong levels of RELT expression within macrophages of lymph nodes, in addition to germinal cells and endothelial cells.

A separate study indicated higher levels of RELL2 expression are a potential risk factor for leukemia [77]. Higher expression of RELL2 along with nine other genes including the mitochondrial enzyme Enoyl-CoA hydratase (ECHDC3) was correlated with poor prognosis and decreased survival in patients with non-acute promyelocytic leukemia. RELL2 expression appeared to be correlated with ECHDC3 in this study, and data suggest that ECHDC3 may increase the chemoresistance of leukemia.

### 9.11. Colorectal Cancer

RELL1 is associated with a ceRNA network and is considered one of several hub genes to be pro-tumorigenic in colorectal cancer [78]. Bioinformatic analysis indicated that RELL1 contributed to the dysregulation of several genes in colorectal cancer by competing for miRNA molecules.

### 9.12. RELL2 Involvement in Additional Cancers

Curiously, although experimental evidence demonstrates RELL2 possesses anti-tumorigenic activities in ESCC, PDAC, and breast cancer cell lines, bioinformatic analysis indicates that RELL2 expression is associated with poor outcomes in several cancers [83]. RELL2 was expressed at higher levels in 16 out of the 20 cancers analyzed, with the highest expression levels in rectum adenocarcinoma and HNSC. Expression of RELL2 was associated with decreased overall survival, disease-specific survival, and/or progression-free interval for several cancers, including adenoid cystic carcinoma, kidney chromophobe (KICH), and uterine carcinosarcoma. Similarly, higher RELL2 expression was found to correlate with more advanced stages of cancer, higher tumor mutational burden, and/or microsatellite instability in several cancers such as colon adenocarcinoma (COAD). Likewise, expression of RELL2 was correlated with neoantigen expression and defects in DNA mismatch repair, and the expression of several methyltransferases. Interestingly, RELL2 expression in tumors was associated with the expression of several checkpoint inhibitors in several cancers including TNFRSF25 (DR3). The cancers in which RELL2 expression appeared to correlate the strongest with different checkpoint molecules included KICH, thymoma, and kidney renal clear cell carcinoma. Yet, as mentioned previously, higher expression of RELL2 predicted better outcomes for patients with PAAD. Although this bioinformatic data do not always indicate causation, there remains the possibility that RELL2 may function in a pro-tumorigenic manner in some cancers, and in an anti-tumorigenic manner in separate cancers. Finally, the mouse ortholog of RELL2 promoted the adhesiveness of several cancer cell lines [17], most notably for the osteosarcoma cell line MG63, consistent with RELL2 functioning to inhibit the migration of tumor cells as was reported for breast cancer cells as discussed previously [62].

## 10. Microbial Infections and Inflammation

RELT was originally identified as a protein that might promote inflammation, as the ECD of RELT stimulated T-cell proliferation in vitro [7]. Yet as described in Section 5.1, the phenotype of mice lacking RELT indicate that RELT functions to inhibit both CD4^+^ and CD8^+^ T cells, MDSCs, in addition to inhibiting the secretion of IFN-γ and TNF-α from T cells [12]. Collectively, the mouse knock-out studies implicate RELT as having an inhibitory effect on the immune system that might be more pronounced in a weak immune response than severe inflammation, as RELT expression in T cells was more sustained in a weak response [12]. Yet as the ECD of RELT has been proven to have independent functions from the rest of the RELT protein [22], an opposing pro-inflammatory function of the ECD of RELT as proposed by Sica et al. may be compatible with an anti-inflammatory function of intact RELT. Studies of point mutations in RELT carried in human families indicate that some [13], but not all [86], of children that inherited point mutations in RELT suffered a history of sore throats or febrile illnesses during childhood.

Autoantibodies against RELL2 were unexpectedly observed in many patients with hypomorphic RAG 1/2 mutations, and these autoantibodies targeting RELL2 may potentially contribute to the inflammation observed in patients with Omenn syndrome [87]. It is not clear how hypomorphic mutations in the RAG1 and RAG2 genes could lead to autoantibodies against RELL2, yet this may be due to a general dysregulation of the humoral immune system by T lymphocytes.

### 10.1. Innate Immune Signaling

RELT likely impacts macrophage responses to TLR4 signaling, as RELT physically interacts with the heat shock protein Hsc70 in a manner that is dependent on prior exposure of macrophages to lipopolysaccharide [88]. Knockdown of RELL1 expression prevented phosphorylation of IκB, p65, and IKK-α in response to TLR2 stimulation, the receptor for *M. tuberculosis* [28], implicating RELL1 as functioning downstream of TLR2.

### 10.2. Regulation of Virus Infection

Proteomic analysis of plasma proteins in an elderly male population indicates that RELT is significantly upregulated in people living with HIV (PLHIV) versus healthy controls when adjusted for age, sex, and smoking status [89]. RELT was upregulated in Langerhans cells and macrophages of PLHIV while immunophenotyping revealed an association of RELT with B cells and neutrophils. It is not clear if these changes in RELT expression in PLHIV are due to the immune response to the virus or are indirectly caused by the long-term administration of antiviral therapy. Due to the ability of RELT to inhibit T cells [12], it is interesting to speculate whether differing expression levels of RELT influence the outcome of HIV infection, especially considering the RELT-binding protein PLSCR1 also binds CD4 and has been implicated in HIV entry [90].

A significant decrease in RELL1 expression in Treg cells lacking the Type I IFN receptor was identified using microarray analysis, implying that RELL1 expression is enhanced by Type I IFN signaling [38]. The ability of Type I IFNs to combat acute viral infections is well established, yet there is some evidence that Type I IFN signaling promotes immune suppression in response to chronic viral infections. Two separate groups demonstrated that RELL1 expression is suppressed by the cytomegalovirus (CMV) protein pUL138, a protein that promotes CMV latency. RELL1 protein expression at the lipid membrane, and to a lesser extent RELT, were both inhibited by the expression of pUL138 in undifferentiated myeloid cells [28]. pUL138 was also shown to inhibit RELL1 expression at both the mRNA and protein level in fibroblasts, in addition to inhibiting viral replication, consistent with RELL1 being silenced during CMV latency [91].

### 10.3. Regulation of Parasite Infection

The RELT promoter region in primary immune cells is hypermethylated in response to helminth infection [92], which is consistent with RELT functioning to inhibit T-cell responses to infection. Considering that T cells lacking RELT overexpress IFN-γ [12], it is unlikely that RELT expression is silenced by helminths due to RELT being a Th1-specific protein, a general suppressive effect of RELT on T-cells as proposed by Choi et al. is more likely [12].

### 10.4. Regulation of Bacterial Infection

RELL1 overexpression enhances secretion of TNF-α, IL-6, and NF-κB from macrophages infected with *M. tuberculosis* or treated with BCG vaccine [28], and as described in Section 8.2, RELL1 promotes *M. tuberculosis* survival in macrophages by inhibiting autophagy. A SNP located most proximally to RELL1 was one of two SNPs to be most associated with persistent and intermittent nasal carriage of S. aureus in one population [93]. Finally, diabetes increases the risk of bacterial infections, and GWAS analysis for missense mutations identified RELL1 as one of two genes most likely mutated in a population of diabetic patients that purchased antibiotics [94], implicating RELL1 as being associated with both diabetes and bacterial infections.

## 11. Other Functions Associated with RELTfms

### 11.1. Enamel Development

An unexpected finding of RELT is that it is essential for proper enamel development. Mutations in several genes are associated with amelogenesis imperfecta AI, and the resulting defects in enamel development result in teeth that are small, discolored, grooved, and prone to rapid wear and breakage. Whole exome sequencing (WES) of individuals with AI from three consanguineous Turkish families identified mutations in the RELT gene [13]. A deletion at position ΔP390, the missense mutation R422P, and a splicing junction mutation in intron 3 were identified in this original study. Subjects with the ΔP390 and R422P mutations were reported to have a history of throat infections and febrile convulsions, respectively [13], which is intriguing considering the high levels of RELT expression within the hematopoietic system. RELT mRNA expression at day 5 was confirmed in odontoblasts and ameloblasts of mice, yet RELT expression in ameloblasts was decreased by day 12 as ameloblasts matured, consistent with RELT contributing to enamel development. Additional missense mutations of RELT were found to be associated with AI in a separate study [86], WES analysis revealed that three families with suspected consanguinity expressed the same T55I point mutation, and a fourth family contained a R422W mutation. In contrast to the earlier study, none of these families reported a history of febrile illness in the afflicted individuals. Interestingly, when considering both studies, three out of the four confirmed mutant proteins in RELT associated with AI either have point mutations at the evolutionarily conserved R422 position or result from a truncated protein (ΔP390), implying that the carboxy terminus of RELT is required for proper enamel development. Mice containing the ΔP390 mutation at both alleles of RELT were created, and no effects of this mutation were observed on the weight of the mice, though they were raised in a sterile environment [13]. The enamel from these mice was hypomineralized, especially near the dentin–enamel junction. Cleavage of the ECD of RELT by the metalloproteinase ADAM10 (Figure 1) was subsequently shown to be required for enamel development in mice [22]. Both ADAM10 and RELT exhibited similar expression profiles in ameloblasts during their development, and ADAM10 is required for the migration of developing ameloblasts. Therefore, the cleavage of the RELT ECD may be important for cell migration during enamel formation. Interestingly, the HPA indicates ADAM10 is expressed in multiple other tissues, and it is interesting to speculate whether the RELT ECD produced by ADAM10 has additional physiological implications besides enamel development. Murine RELL2 is also expressed in the inner enamel epithelium of developing mice [17], suggesting that RELL2 may also function in the development of enamel.

### 11.2. Reproduction and Development

Multiple lines of evidence indicate that RELTfms are important for processes associated with reproduction and development, in addition to the development of enamel. RELL1 was identified within a quantitative trait loci (QTL) associated with a higher number of stillbirths in domestic pigs [95]. Knockdown of expression of the cd26 peptidase resulted in a more than two-fold reduction in RELL1 expression in developing blastocytes as determined by RNAseq analysis [96]. cd26 is a marker for embryo implantation, and a reduction in cd26 expression is associated with decreased development of parthenogenetically activated porcine embryos. These separate studies in pigs demonstrate that RELL1 is likely an important gene for embryonic development. An SNP highly associated with early puberty of a tropical species of cattle (*Bos indicus*) was located most proximally to the RELL1 gene [97], and RELL1 was associated with prolificacy in goats in a separate study [98], further supporting a role of RELL1 for development. Knocking down expression of the transcription factor ZBED1 results in both increased apoptosis, and increased expression of several genes including RELT, in a choriocarcinoma cell line induced to differentiate into trophoblasts [60]. These results indirectly imply a role for RELT in promoting apoptosis in both trophoblasts as well as cancer, as the BeWo cell line used in this study is a carcinoma cell line.

### 11.3. Blood Pressure and Myocardial Infarction

The SNP rs75612301 associated with high blood pressure was positively correlated with RELT expression [99], though a direct correlation between RELT and high blood pressure is currently lacking. Previous studies indicated that circulating RELT levels are altered by heparin treatment during catheterization (*p* = 0.025) yet not by myocardial injury [100]. Of note, circulating RELT antigen in the blood was much higher in females than in males in this study (1.5 × 10^−27^), though the significance of this is unclear. RELT was significantly associated with the incidence of myocardial infarction and cardiovascular disease in elderly males from one study [101]. RELT was one of six genes most associated with the development of cardiovascular disease in a 5-year follow-up of persons living with HIV [89]. It is worth noting that modest levels of RELT protein were detected in the kidney [21] and it is therefore possible that RELT influences hypertension, and the risk of myocardial infarction, through its interaction with the OXSR1 [9] and SPAK [27] kinases, as both of these kinases are targets for the treatment of hypertension based on their ability to regulate ion transporters in the kidney [102]. As mentioned previously, circRELL1 promotes inflammation in response to oxidized LDL [18], providing another connection between RELTfms and cardiovascular disease.

### 11.4. Brain and Behavior

As discussed in Section 5.2, the most pronounced phenotype associated with mice lacking RELL1 was associated with behavior and auditory processing [30] and evidence indicates that RELL2 may also impact behavior. The SNP rs14521 resulting in a missense mutation (C→A) within an exon of the RELL2 gene is significantly higher in patients with methamphetamine dependence in comparison to control patients [103]. This SNP resulted in decreased expression of RELL2 in a variety of tissues including the brain, yet it is not clear what effect this mutation has on the function of RELL2 protein, or the potential significance of this SNP to brain-related disorders. It should be noted that rs14521 influenced the expression of additional genes in the vicinity of RELL2 on 5q31.3, as it resulted in reduced expression of PCDHGB6, and increased the expression of FCHSD1. There are currently no additional studies suggesting a link between RELL2 and neurological disorders, yet it should be noted that the HPA predicts a higher expression of RELL2 in the brain than either PCDHGB6 or FCHSD1. Although a mouse knockout of the RELL2 gene has not yet been reported, the dramatic impact of RELL1 loss on the hearing and behavior of mice indicates that RELTfms can function to affect neuronal networks and human behavior.

## 12. Additional Binding Partners of RELTfms

### 12.1. Hsc70

RELT physically binds the heat shock protein Hsc70 (Figure 1), yet only co-IPs with Hsc70 in monocyte extracts (RAW264.7) that were treated with LPS [88]. Hsc70 enhances the TLR4 signaling pathway in RAW264.7 cells treated with LPS, suggesting that RELT may participate in the TLR4 signal transduction pathway. Although heat shock proteins are best characterized by helping misfolded proteins find their proper conformation, Hsc70 is involved with lysosomal targeting, vesicle coating, and delivery of cytosolic proteins to the nucleus and other compartments. It would therefore be interesting to test whether Hsc70 alters the cellular localization of RELT, as the HPA predicts that RELT is localized in the nucleus.

### 12.2. PLSCR1

RELTfms bind the protein PLSCR1 [25] PLSCR1 can be phosphorylated by OXSR1 in vitro, yet only if RELT was also transiently transfected, indicating that RELT may be simultaneously bound by OXSR1 and PLSCR1 as a signaling complex [25]. The impact of PLSCR1 on RELT-induced death in 293 cells was inconclusive, yet an interesting observation was made when RELT and PLSCR1 were both co-transfected in 293 cells. Recombinant PLSCR1 appeared to adopt the cellular localization of RELT when RELT was co-transfected, indicating that RELT may alter the localization of PLSCR1 to subcellular vesicles. PLSCR1 was originally identified to scramble phospholipids at the surface of the plasma membrane [104], an important step in the clearance of apoptotic cells, as phagocytic clearance of apoptotic cells requires externalized phosphatidylserine [105]. PLSCR1 is now recognized to also be a transcription factor that can upregulate the expression of the inositol 1,4,5-triphosphate receptor [106]. Additionally, PLSCR1 activates apoptosis in several cell types [107,108,109] and is an IFN-stimulated gene that itself enhances antiviral responses [110], which is of interest given the established relationship between IFN signaling and both RELT and RELL1. PLSCR1 expression levels are a prognostic indicator for the survival of LAML patients [111] and PLSCR1 promotes the differentiation [112] and apoptotic death of leukemic cells [113]. Conversely, PLSCR1 was reported to promote tumorigenesis in both breast cancer cells [114] and colorectal carcinomas [115], indicating that the impact of PLSCR1 on cancer may differ depending on the type of cancer. RELL1 inhibits autophagy [28] and also binds PLSCR1, and it is interesting to note that PLSCR1 also acts as an inhibitor of autophagy in both lymphoma [116] and leukemia cells [117].

### 12.3. MDFIC

Finally, all three RELTfms were found to bind and co-localize with MDFIC (also referred to as HIC), a transcription factor that differentially regulates the HIV and HTLV-1 genomes [118] and is also implicated in many cancers. A truncated mutant of RELT containing only the first 252 amino acids of RELT, corresponding to the first 61 amino acids of the ICD, co-immunoprecipitated with MDFIC, suggesting that MDFIC interacts with RELTfms in regions of the ICD proximal to the plasma membrane. Similar to RELT, MDFIC is expressed at high levels in the immune system [119]. The effect of MDFIC on cancers may vary, as it enhances chemoresistance of cancer stem cells [120] yet suppressed the growth of colorectal cancer cells [121]. The MDFIC gene is located most proximally to a deletion of chromosome #7 commonly lost in LAML patients [122], which is of interest due to the correlation of RELL2 with LAML [77,83] and the demonstrated anti-leukemic properties of the RELTfm-binding protein PLSCR1 [111,112,113]. More studies are clearly needed to elucidate the physiological significance of MDFIC binding to RELTfms.

## 13. Conclusions and Future Directions

Accumulating evidence indicates that RELTfms make a significant impact on many diverse processes and play a vital role in both cancer and the regulation of the immune system. Future studies are needed to identify the ligand for RELT, and to further explore the correlation between RELT and an immunosuppressive environment. Since RELTfms were named for their abundant expression in the hematopoietic system, a thorough understanding of a RELTfm with a specific type of cancer will need to take into account the activities of RELTfms in the immune response directed against the cancer, in addition to the effect of RELTfm expression within the malignant cells.

RELT is most homologous to the TNFRSF members TROY (TNFRSF19), DR3 (TNFRSF25), OX40 (TNFRSF4), and LTβR (TNFRSF3) in the ECD. TROY is highly expressed in embryonic development, is expressed at high levels in the brain and skin, activates NFκB [123], and is associated with glioblastoma invasion, survival, and chemoresistance [124]. Like RELT, there is no identified ligand from the TNFSF that binds TROY, yet TROY has been reported to promote tumorigenesis through its interaction with the TGFβ receptor [125]. RELTfms likewise may potentially physically bind and modulate other signal transducing networks, regardless of whether a ligand specific for RELT exists. The TNFSF ligands for DR3 are TL1A and TWEAK; DR3 is similar to RELT in that it is expressed at highest levels in the hematopoietic system and can activate either apoptosis or activation of NFκB [126]. DR3 activation in T cells by TL1A increases IL-2 responsiveness and T cell proliferation in response to TL1A [127], which are opposite the effects of RELT based on the mouse knockout phenotype [12]. Yet DR3 is also important for T cell death, and lack of DR3 impairs negative selection during thymic development [128]. This serves as an important reminder that reports of RELT’s ability to induce either death or promote survival through NFκB are not necessarily in disagreement with each other, as these opposite outcomes may be cell or context-dependent. OX40 binds the TNFSF member OX40 and is important for T cell activation, as mice or humans lacking OX40 displayed defective formation of CD4^+^ effector memory cells [129,130]. Like RELT, OX40 stimulates NFκB activation [131], yet in contrast to RELT, OX40 functions as a co-stimulatory molecule essential for the survival of activated T cells [132]. LTβR binds to the TNFSF members LTβ and LIGHT, and LTβR activation by LTβ is essential for proper lymphoid organ development [133]. LTβR plays an important role in Type I IFN responses and mice lacking the receptor exhibit defective antiviral immunity [134]. Finally, LTβR can activate NFκB and stimulate pro-inflammatory cytokine secretion, and the ability of LTβR to promote inflammatory cytokine secretion and tertiary lymphoid tissues may increase the risk of cancers in the liver and prostate [135]. Much less is known about RELT in comparison to other TNFRSF members, and more research is needed to understand the relationship between RELTfms and diseases associated with cancer, microbial infection, inflammation, and development. In summary, RELTfms are associated with various cancers, playing roles in tumorigenesis, the immune response to tumors, and prognosis prediction. Additionally, RELTfms have demonstrated activities in cell death pathways, development, inflammation, microbial infections, and behavior. With continued exploration, the diverse functions of these evolutionarily conserved proteins will offer new avenues for therapeutic targeting and clinical management of different diseases.

## Figures and Tables

**Table 1:** Summary of associations between RELTfms and different cancers.

Cancer or Cancer Cell Line	Gene or Protein	Predicted Effect, Prognostic Indicator, or Expression Level	Details of Study Implicating RELTfm with Cancer	Reference
Breast cancer	RELT	Autoantibodies against RELT potential biomarker for cancer	RELT likely upregulated or altered in breast cancer, based on the identification of RELT autoantibodies in serum of women with breast cancer, that are absent in healthy controls	[65]: Zhong L. et al., 2008
Breast cancer	RELT	Upregulated in cancer	Upregulated *RELT* RNA in mouse mammary EpRas tumor cells treated with TGF-β for two weeks to induce EMT. RELT associated with cluster of genes upregulated in human triple-negative breast cancer, based on microarray analysis of 38 breast cancer cell lines and 107 human breast cancer tissues.	[66]: Johansson J. et al., 2015
Breast cancer	RELL2	Protective against cancer	Expression of RELL2 inhibits migration and invasion capabilities of breast cancer cell lines 4T1 and MDA-MB-231 (231) ex vivo. The saponin PP-VI indirectly upregulates RELL2 expression, and PP-VI treatment inhibited growth of 4T1 in mice in vivo and induced cell cycle arrest and apoptosis of 4T1 and 231 cells ex vivo.	[62]: Wang P. et al., 2019
ESCC	RELT	Pro-tumorigenic, Poor prognostic indicator, Upregulated in cancer	RELT functions as an oncogene that upregulates NF-κB and inhibits pathways based on both ex-vivo and in-vivo studies. RELT expression upregulated ESCC based on bioinformatic and laboratory studies. High levels of RELT expression serves as a poor prognostic indicator for survival of ESCC patients.	[29]: Yao W. et al., 2021
ESCC	RELL2	Protective against cancer, expression decreased in ESCC cell line.	RELL2 expression protective against ESCC both in vivo and ex vivo. Endogenous expression of RELL2 is low in tumorigenic ESCC cell line. Report also highlighted alternative splicing of RELL2 hnRNA that can lead to nonsense mediated decay message, and less RELL2 protein expression, in ESCC cell line.	[67]:Tang S.J. et al., 2020
Prostate cancer	RELT	Poor prognostic indicator, Upregulated expression	Upregulated RNA in cancer compared with non-malignant prostate tissue, expression increases with advanced staging. Bioinformatic analysis indicates higher RELT expression is poor prognostic indicator for prostate cancer patients.	[68]: Ge S. et al., 2021
Prostate cancer	RELT	Poor prognostic indicator	RELT a member of a gene signature, SigIQGAP3NW, that predicts poor outcomes in prostate cancer. Bioinformatic analysis indicates RELT expression associated with an immunosuppressive environment and poor outcomes in prostate cancer.	[69]: Mei W. et al., 2023
Glioma	RELL1	Poor prognostic indicator, Upregulated expression	Bioinformatic analysis indicates RELL1 RNA is upregulated in glioma and correlates with more severe cases of glioma and an immunosuppressive environment. Expression of RELL1 inversely correlated with NK cell activity.	[70]: Jin X. et al., 2020
Glioblastoma	RELL1	Potentially pro-tumorigenic	RELL1 containing a N255D mutation in intracellular domain only expressed in glioblastoma cell lines, not in normal astrocyte cell lines.	[71]: Rose M. et al., 2021
Gastric cancer	RELL1 circRNA	Protective against cancer, Downregulated expression in cancer	Demonstrated that circRELL1 expression inhibits gastric cancer both in vivo and ex vivo. Propose that circRELL1 protects against cancer by sponging pro-tumorigenic miR637. Exosomal circRELL1 downregulated in gastric cancer.	[19]: Sang H. et al., 2022
PDAC	RELL2	Anti-tumorigenic, Good prognostic indicator	RELL2 induces apoptosis of AsPC-1 cell line. Splicing factor DHX38 required to remove intron 4 of RELL2 transcript to avoid nonsense mediated decay of RELL2 transcript. Higher levels of RELL2 expression a good prognostic indicator.	[72]: Li Z. et al., 2023
Lung (NSCLC)	RELT	Upregulated	RELT RNA upregulated 2.1-fold in lung cancer in tumors of 4 human patients, most patients had NSCLC. RELT expression downregulated 1.9-fold in response to romidepsin treatment in at least one patient with lung cancer.	[73]: Schrump D. et al., 2008
Lung	RELT	Upregulated	RELT protein upregulated in mouse lung tumors 2-fold. Tumorigenesis in mouse lungs induced by K-ras G12D mutation.	[26]: Jung H. et al., 2019
Lung (SCLC)	RELT	Good prognostic indicator	RELT expression expressed at higher amounts in low-risk group for SCLC.	[74]: Zhang Z. et al., 2021
Renal cancer	RELT	Poor prognostic indicator	Bioinformatical data indicates RELT expression associated with poor survival, RELT expression may be correlated with an immunosuppressive environment.	[75]: Cui Y. et al., 2022
Head and neck cancer	RELT	Poor prognostic indicator	RELT expression inversely correlated with overall patient survival. Bioinformatic data indicates RELT expression associated with immunosuppression markers, and that RELT expression is downregulated by several chemotherapeutic agents.	[76]: Guo Y. et al., 2021
B cell lymphoma	RELT	Upregulated	RELT protein expression upregulated in B cell lymphoma biopsies versus healthy lymph nodes.	[23]: Cusick J. et al., 2020
Non-acute promyelocytic leukemia	RELL2	Poor prognostic indicator	RELL2 is one of 9 genes whose expression was correlated with poor survival of patients with non-acute promyelocytic leukemia.	[77]: Zhao Y. et al., 2022
Colorectal cancer	RELL1	Predicted to be pro-tumorigenic	Bioinformatic analysis indicates RELL1 may contribute to dysregulation of gene expression in colorectal cancer by competing for miRNA molecules.	[78]: Kadkhoda S. et al., 2021

A summary of manuscripts highlighting associations with different cancers is presented. Abbreviations: esophageal squamous cell carcinoma (ESCC), pancreatic ductal adenocarcinoma (PDAC), non-small-cell lung cancer (NSCLS), and small-cell lung cancer (SCLC).

## Data Availability

Not applicable.

## Supplementary Materials

The following supporting information can be downloaded at: https://www.mdpi.com/article/10.3390/biomedicines11102695/s1, Figure S1: Expression of RELT in different cell lines. Figure S2: Expression of RELT in different tissues. Figure S3: Expression of RELL1 in different cell lines. Figure S4: Expression of RELL1 in different tissues. Figure S5: Expression of RELL2 in different cell lines. Figure S6: Expression of RELL2 in different tissues.
