# Supplementary material for: The RELT Family of Proteins: An Increasing Awareness of Their Importance for Cancer, the Immune System, and Development
Source: Biomedicines. 2023 Oct 2;11(10):2695. doi: 10.3390/biomedicines11102695 (PMC10603948; doi:10.3390/biomedicines11102695)
Supplement: Supplementary file 1 [file biomedicines-11-02695-s001.zip › biomedicines-2585491-supplementary.pdf]

biomedicines

Review

The RELT Family of Proteins: An Increasing Awareness of Their Importance for Cancer, the Immune System, and Development

Supplemental Figures S1–S6: Expression of RELTfms in cell lines and tissues. Gene expression data for RELT/RELL1/RELL2 in cell lines and tissues are exported from the "All RNA-seq and ChIP-seq sample and signature search" (ARCHS[4]) platform (https://maayanlab.cloud/archs4/). The ARCHS[4] platform is a resource that provides mas-sive mining of publicly available gene and transcript data derived from all human and mouse RNA-seq studies available on the Gene Expression Omnibus (GEO) and the Se-quence Read Archive (SRA). Lachmann A, Torre D, Keenan AB, Wang L, Silverstein MC, Ma'ayan A. Massive mining of publicly available RNA-seq data from human and mouse. Nature Communications 9. Article number: 1366 (2018), doi:10.1038/s41467-018-03751-6

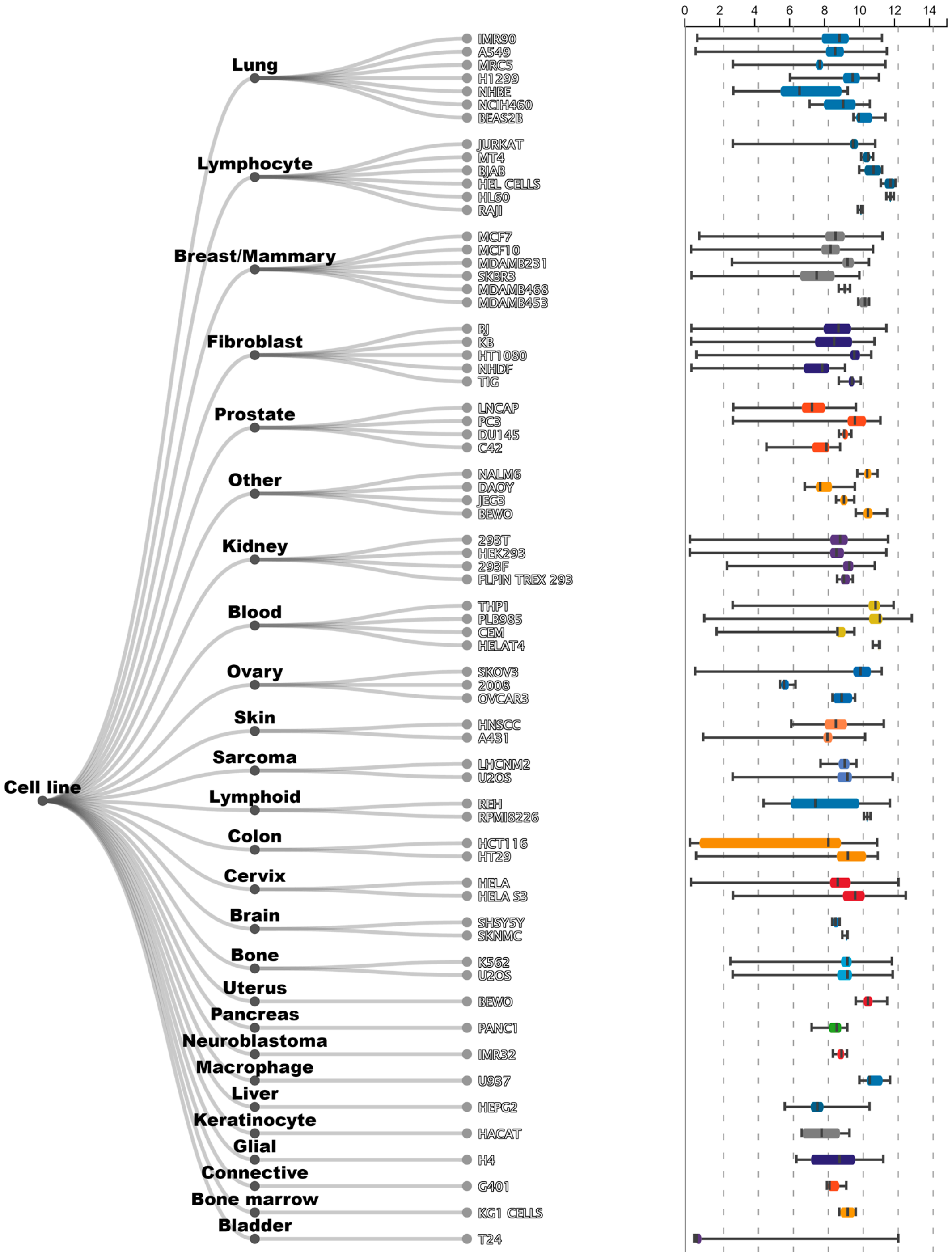

Supplemental Figure S1. Expression of RELT in different cell lines.

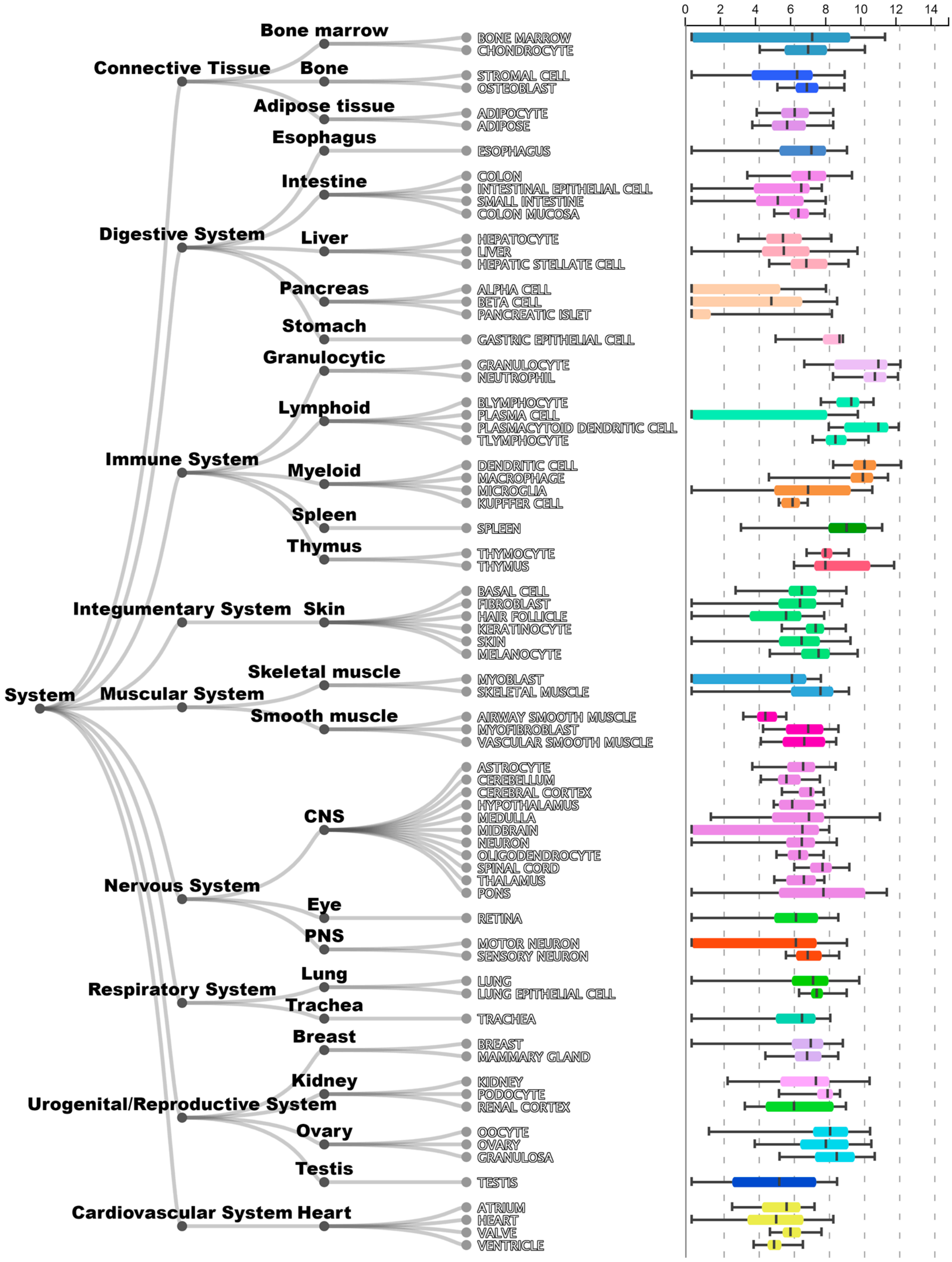

Supplemental Figure S2. Expression of RELT in different tissues.

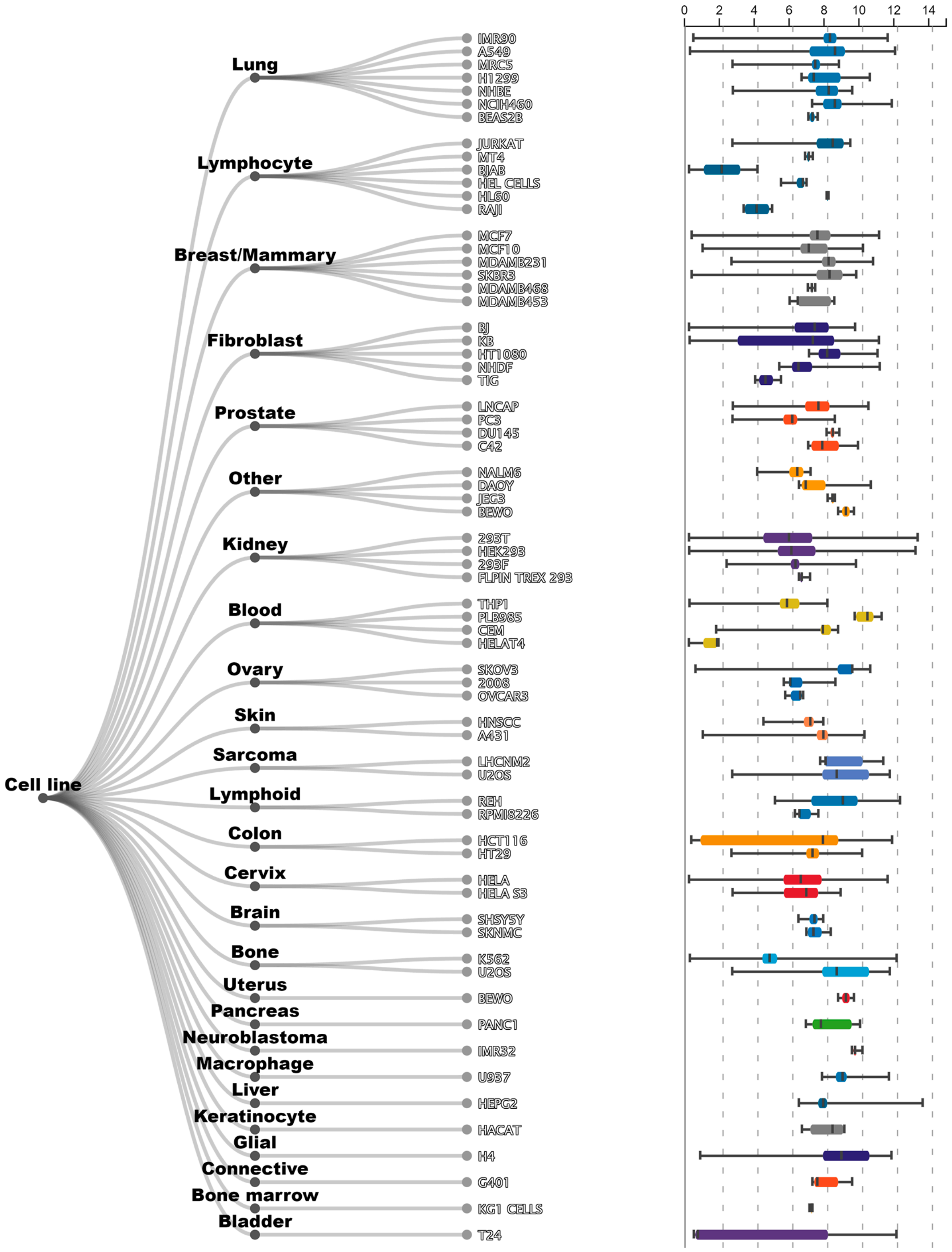

Supplemental Figure S3. Expression of RELL1 in different cell lines.

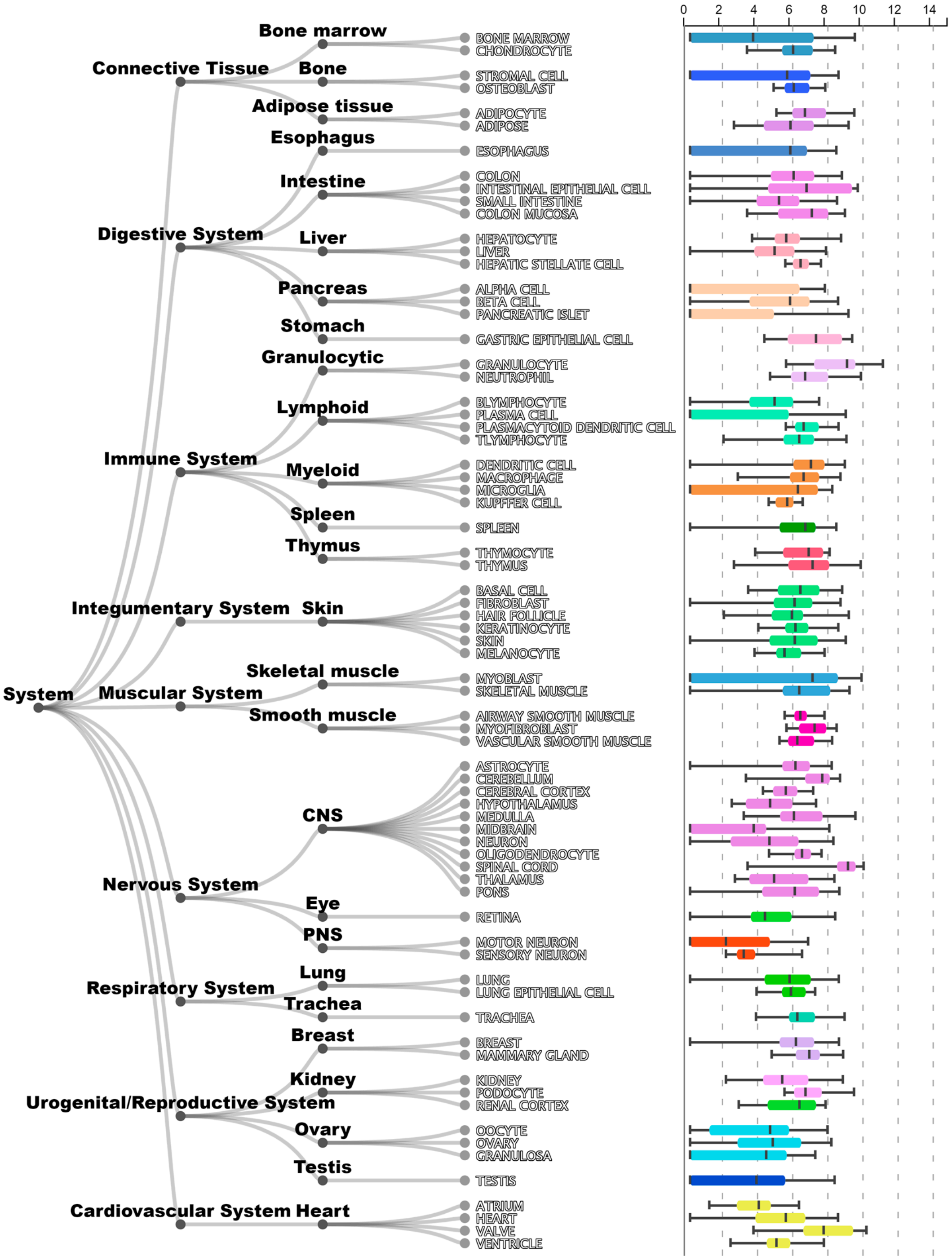

Supplemental Figure S4. Expression of RELL1 in different tissues.

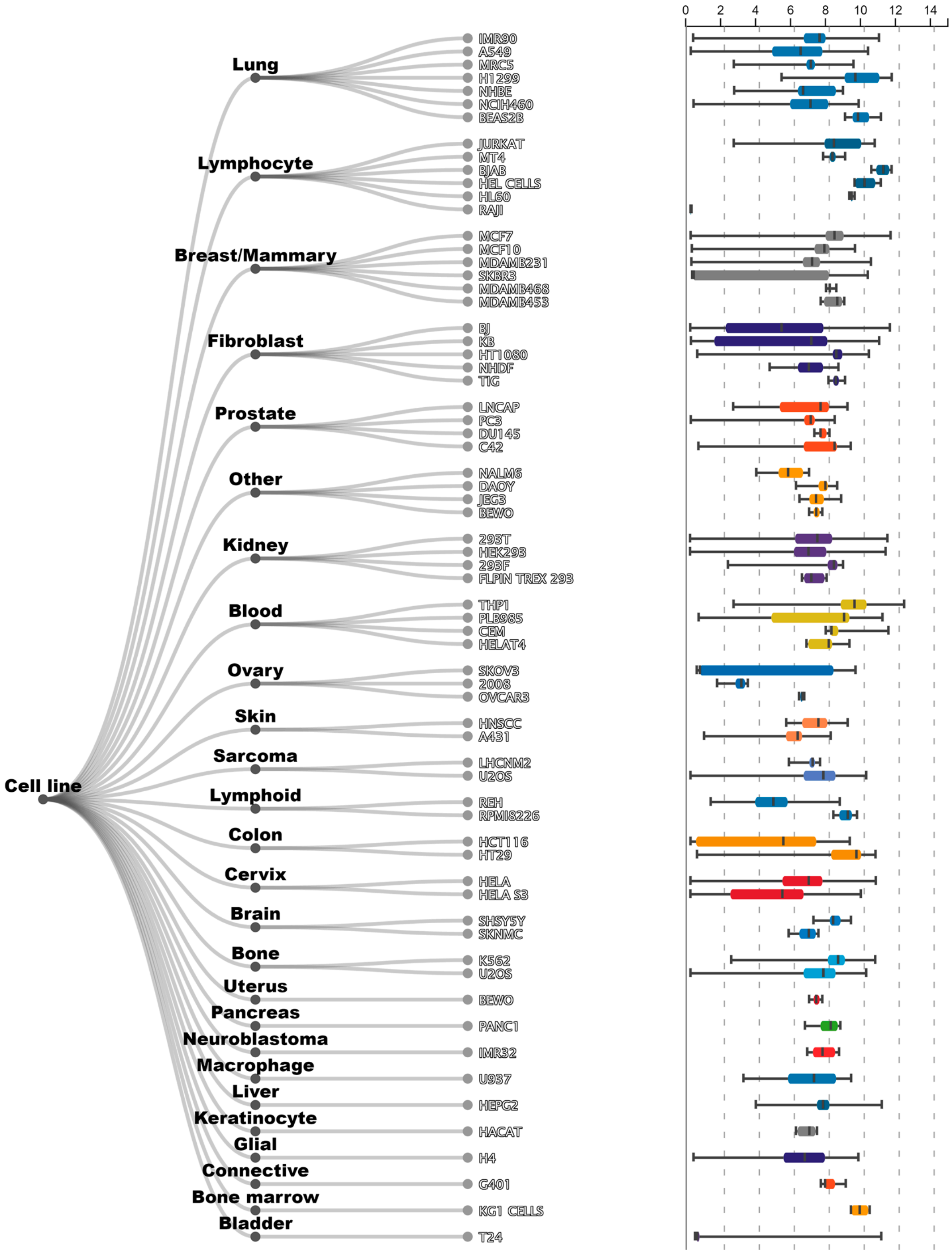

Supplemental Figure S5. Expression of RELL2 in different cell lines.

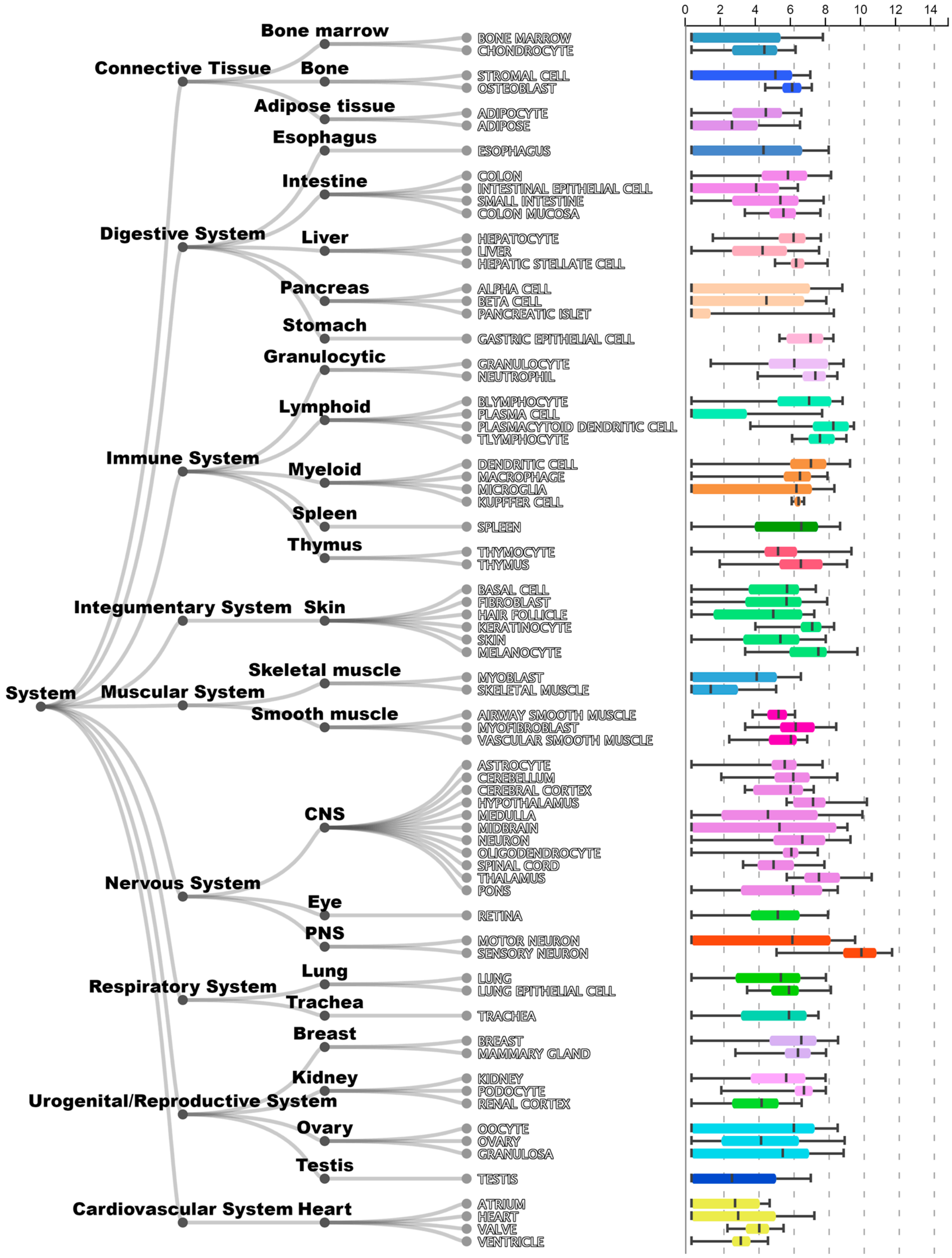

Supplemental Figure S6. Expression of RELL2 in different tissues.
